# Visual analytics of route recommendation for tourist evacuation based on graph neural network

**DOI:** 10.1038/s41598-023-42862-z

**Published:** 2023-10-11

**Authors:** Lin Zhang, Jifeng Xu, Xiaotian Pan, Jianing Ye, Weijie Wang, Yanan Liu, Qian Wei

**Affiliations:** 1 School of Information Management and Artificial Intelligence, Zhejiang University of Finance and Economics, Hangzhou, 310018 China; 2 The Smart Oilfield Business Department of Kunlun Digital Intelligence Technology Co., Beijing, 102206 China

**Keywords:** Computational science, Computer science

## Abstract

The overcrowding of scenic spots not only threatens tourists’ safety but also affects the travel experience. Traditional methods for addressing tourist overload have involved limited access and guided evacuation. While limited access has been effective, it often results in a diminished tourist experience. Moreover, the existing guided evacuation rarely considers the impact on tourists’ experience, resulting in a low willingness to cooperate and making it difficult to estimate evacuation effort efficiency. To solve these problems, this paper proposed a tourist evacuation route recommendation algorithm based on a graph neural network considering the similarity of tourism styles (PER-GCN) and designed a visualization system to simulate and analyse evacuation efficiency. First, the interaction matrix of tourists and scenic spots was constructed using graph mining to extract the high-order interaction information. In the output layer, the similarity between scenic spots and tourism styles was calculated to further improve the accuracy of scenic spot recommendations. Second, due to route complexity and the real-time carrying capacity of scenic spots, the researchers optimized the evacuation routes. Finally, taking the West Lake spot as the case study, the effectiveness of PER-GCN was verified. Additionally, a visualization system was designed to monitor tourist flow in real time and analyse tourist portraits according to the clustering results of scenic spot styles. In addition, the evacuation efficiency of scenic spots was analysed by adjusting the parameters of tourists’ willingness to cooperate, evacuation batch, and the weight of route complexity and scenic spot carrying capacity.

## Introduction

Along with the rising economy, tourism is increasing the demand for people to improve their quality of life. Tourists’ safety is the primary factor affecting tourism development. However, due to the seasonal and concentrated characteristics of tourism activities, the overload threatens tourists’ safety. For example, in Itaewon, South Korea, a stampede occurred during the 2022 Halloween festival, causing hundreds of tourist casualties. The incident exposed the shortcomings of tourism management in flow warnings and tourist evacuation. As a representative of Hangzhou (China) scenic attractions, West Lake is often overcrowded during the tourism season. In addition, there are great differences in tourist distribution. For example, “White Causeway”, “Su Causeway”, and other popular scenic spots are overloaded, while the surrounding scenic spots with similar styles have low visitor traffic. The uneven distribution in space-time further increases management difficulty and leads to a poor tourist experience.

The traditional methods for solving overcrowded areas include limited access and guided evacuation.Although the former has higher execution efficiency, the latter is more reasonable and humanized^1^. However, designing an efficient and feasible evacuation method is key to solving the overcrowding problem. Therefore, many studies began to be engaged in mining tourists’ behaviour and road features to improve the diversity and rationality of evacuation routes. For example, Karthik and Suja^2^ proposed an evacuation method based on pedestrian familiarity with the location. Some studies consider tourists’ walking habits to design evacuation models and route planning, such as body-turning behaviour^3^, walking on the right^4^ and travel behaviour^5^. However, the above methods do not account for tourists’ travel preferences. Therefore, the evacuation destination cannot meet the travel needs of tourists and reduces their willingness to cooperate. Although there have also been some studies on personalized travel recommendations, they aim to plan a trip without considering tourist flows. For example, Paulavicius et al.^6^ considered start and end locations and tourist preferences and limited the time spent on similar attractions. Nagarajan et al.^7^ used a travel review dataset to classify traveller ratings of travel destinations. Based on that, this paper proposes a personalized scenic spot recommendation method to alleviate overcrowding, considering more factors, such as tourist travel style, scenic spot carrying capacity, and route complexity.

Recommendation algorithms can help to exploit the preferences of tourists and realize personalized recommendations.Recommendation algorithms can be roughly divided into two categories: traditional recommendation algorithms and recommendation algorithms based on deep learning networks. The traditional recommendation algorithm uses machine learning methods to calculate the matching degree or similarity degree of the items to be recommended based on user characteristics or item characteristics, which can be divided into content-based, user-based, collaborative filtering, and mixed recommendation. Recommendation algorithms based on deep learning networks are better at processing big data and mining complex structural information. For example, graph neural networks (GNNs)^8^ can process graph structure information, capture higher-order relationships through information transfer between graph nodes, and have a stronger interpretation ability; therefore,many researchers use GNNs and their variants to design recommendation algorithms. For example, Wang et al.^9^ proposed a top-N personalized recommendation with a graph neural network (TP-GNN) in MOOCs to learn the explicit representation of the structural relation of items. For trip recommendations, GNNs have shown good performance in travel package recommendations^10^, travel mode recommendations^11^ and popular attractions for travellers based on seasons and tourists’ interests^12^. However, the above topics are not intended for evacuation purposes. Therefore, this paper designs a scenic spot recommendation method for evacuation based on GNNs and further improves GNN accuracy in predicting attractions of tourist interest..

Based on the above analysis, this research proposed a personalized evacuation route recommendation algorithm based on a GNN, considering tourists’ travel preferences, evacuation route complexity, and the real-time carrying capacity of scenic spots. In addition, taking West Lake as an example, this paper designs a scenic spot visualization system that can monitor the capacity of each scenic spot in real time and carry out evacuation planning for overloaded scenic spots based on the proposed method. The recommendation algorithm and visualization system can also be applied to managing other scenic spots to improve tourist management and provide better service. In summary, the contributions of this paper are as follows:

- To improve GNN accuracy in predicting the attractions of tourist interest, this paper proposes an improved graph convolutional neural network named PER-GCN. In PER-GCN, a tourism style similarity calculation method is designed to obtain tourist preference scores based on mining the interaction matrix between tourists and scenic spots..
- For the purpose of tourist evacuation, this paper proposes a personalized scenic spot recommendation method based on PER-GCN. This paper considers more factors, such as tourist preference, scenic spot carrying capacity, and route complexity..
- A visualization system is designed for West Lake administration. The system can be used to monitor tourist flows in real time and use the proposed method to evacuate tourists in overloaded scenic spots. The efficiency and effectiveness of the evacuations are simulated in this system by controlling different parameter settings.

The rest of this paper is organized as follows. “Related work” section reviews the related works. “The proposed evacuation route recommendation method” section proposes the evacuation route recommendation method. “Introduce the design method of the visualization system” section introduces the system design of the visualization method. “Experimental results and visualization analysis” section describes the data source and visualization task and presents the results of comparative experiments and visual analysis. “Summary and future work” section concludes the study and provides several possible future works.

## Related work

In this paper, the related work includes evacuation management, a collaborative filtering algorithm based on a GNN, a spatial network and model visualization.

### A short review of evacuation management

There are two hot topics in evacuation management, including evacuation factor analysis and evacuation planning. The evacuation factor analysis is related to the effectiveness and efficiency of evacuation plans. In the case of a major emergency, evacuation efficiency is most important, and thus, the decision-maker should consider transporting the stakeholders from the danger area to the refuge within the shortest time^13^. However, in some cases, the situation is not an emergency or can be predicted and planned in advance^14^. For tourist evacuation existing studies on tourist evacuation focus on evacuation efficiency, such as accessibility and connectivity. For example, Gehlot et al.^15^ consider road traffic and arrival time to develop effective evacuation strategies in hurricane disasters, where the preferred destination is decided by the traveller. Ricardo et al.^16^ used site surveys and questionnaire surveys to show a high willingness to evacuate but also found that some travellers want to understand the evacuation direction and the evacuation area location. Therefore, the above research suggests that travellers’ decisions and cooperation are very important, but travellers’ preferences are rarely considered in evacuation plans. In tourism route planning, tourists’ preferences are an important factor in making recommendations for attractions; for example, Zahra et al.^17^ used users’ reviews on tourism social networks to extract their preferences and provide personalized recommendations.Nitu^18^ analysed users’ Twitter data and their friends and followers to understand recent travel interests and design a personalized recommendation model incorporating time sensitivity. However, most studies on tourist interests have not considered the real-time carrying capacity of scenic spots; therefore, they cannot be directly used as a recommended model for tourist evacuation. For this purpose, evacuation efficiency, carrying capacity and tourist interests should all be considered.

The evacuation model design is key to improving the calculation efficiency and achieving the evacuation goals. An appropriate evacuation model can ensure the safety of stakeholders. The principle of the shortest route when evacuating is to plan the shortest escape route according to experience to escape from the danger area in the fastest way.However, the shortest route algorithm is only the best choice for the route, and there are different route planning algorithms for different route planning objectives. Several types of evacuation models exist. For example, Praditya et al.^19^ use the hybrid (collaborative filtering and content-based filtering) method to recommend tourist destinations. Kirchner et al.^20^ introduced a cellular automatic model into evacuation modelling for the first time to build a pedestrian movement migration model. Deep learning models can mine abstract problems through big data learning and model training and rely on the powerful computing power of computers to carry out big data operations, so they are more suitable for analysing big data than traditional evacuation models and computing methods and can show higher data processing capacity and prediction ability. Currently, deep learning models have been widely used in tourism recommendation. For example, He et al.^21^ proposed a travel route recommendation algorithm that combines a convolutional neural network and collaborative filtering. Li et al.^22^ proposed a network evacuation route optimization model based on a heuristic fusion algorithm. Duan^23^ analysed the user’s historical interest from check-in behaviour in detail and constructed a convolutional neural network to extract the potential features of the target visiting area. Then, the user’s active interest is learned from the user’s historical interest. Therefore, using an appropriate deep learning model to explore tourist preferences and then developing an evacuation model is an effective approach.

### Recommendation algorithm based on a graph neural network (GNN) and its application

Based on the complex graph structure, the recommendation algorithm based on GNN can better extract the interactive information between users and items. With its superior performance in feature extraction, applying GNNs in recommendation has become an inevitable development trend. To integrate users’ interests and preferences more effectively and improve accuracy according to different application scenarios, correlation GNNs have also been improved. Wu et al.^24^ roposed a recommendation model (SR-GNN) based on GNN, which extracts short-term dynamic user preferences in sessions and forecasts user behaviours through GNN and traditional attention mechanisms. Cui et al.^25^proposed a nodewise graph neural network (NGNN) model based on the graph attention mechanism in clothing recommendation, using the attention mechanism to calculate graph output and predict compatibility scores to recommend reasonable clothing collocation to users. Zhang et al.^26^ proposed a model combining many graph convolution network (GCNS) encoders/decoders with intermediate supervisors to improve the final prediction performance.

With successful GNN application in the recommendation field, graph neural networks in many fields have also been applied to recommendation systems. Ying et al.^27^ applied a GNN for the first time to solve the problem of industrial web page recommendation. In the face of large-scale web information, compared with the traditional deep learning algorithm, the recommendation results of this model met the needs of users and achieved more satisfactory results. Li et al.^28^ proposed the hierarchical fashion graph neural network (HFGN) model for personalized clothing recommendation. The original historical records were used as users’ personal preferences to model users and clothing, and the personalized recommendation output was predicted and rated after the hierarchical graph convolution. Wu et al.^29^ proposed a session recommendation architecture based on neural networks (SR-GNN) to predict user behaviour through anonymous sessions and combine graph models into presentation session sequences. The model uses an attention mechanism to learn users’ general interests and current interests and make recommendations according to users’ personal interests. In addition, Min et al.^30^ designs a self-supervised Graph neural network model based on the distribution characteristics of power-law distribution. Feng et al.^31^ used graph convolution to calculate the effectiveness of scores to reduce the error in predicting commodity recommendations.

According to our investigation, there is little research on the application of GNNs in evacuation route recommendation. Based on the principle of “no free lunch”, no algorithm is suitable for solving all problems. Since different scenic spots may have similar styles, tourists usually have relatively consistent preferences for different styles of scenic spots. Therefore, this paper proposes an advanced GNN recommendation algorithm based on the scenic spot-style similarity score and applies it to the West Lake tourist evacuation in practice.

### Visualization technology in spatial networks

In recent years, visualization technology has been widely used in many fields, such as network visualization^32^, geospatial data visualization^33^, and model visualization^34^, and network visualization is an important field of information visualization. It displays the relationship between elements in a graphical way to help users observe and analyse data and mine valuable information. Therefore, it has been highly valued by scholars from various countries and widely applied in various network data analysis and management fields^35^ . Zhou et al.^36^ built a network diagram to analyse the mental health status of college students based on the personality quantization table. Zhao et al.^37^ visualized the node-link graph and constructed it as a network graph to express the background story, influencing graph perception and recognition. Among them, spatial network visualization is an important form of network visualization. Zhou et al.^38^ represented human movement data in the form of origin-destination (OD), selected OD flows in the vector representation space and designed a set of visual coding to represent the interaction between OD flows so that reduced visual clutter and enhanced OD flow relevance were realized. Zhou et al.^39^ constructed a matrix to reconstruct the temporal and spatial attributes of taxi OD data and rapidly identified different urban functional areas and analysed crowd flow patterns through the visual interaction framework.

A geospatial map is a kind of image that describes the situation of various resources in different geographical locations or related things. In the scenic spot recommendation process, geospatial location reflects people’s travel choices. Geographic coordinates are often additional attributes of nodes, such as origin and destination. Silva et al.^40^ designs a spatio-temporal visualization of the PM10 concentrations to monitor and understand the behavior of PM10 concentrations in different locations or related things. Wang et al.^41^ proposed a visual analysis system for acquiring traffic bayonet vehicle operation data based on radio frequency identification technology for Nanjing City. The system used dots on the map to describe the geographic spatial location of traffic bayonets and designed attributes such as colour, number, and direction of arrows to represent information such as traffic flow speed, direction, and flow size at different bayonets. Users can intuitively analyse and discover important traffic hubs and traffic flows in Nanjing. In addition, according to the individual attributes of tourists, the target tourists are modelled through the mastered tourist data to provide strong support in solving the overall tour planning of tourist attractions. Zhou et al.^42^ used massive network data to construct tourist portraits of Shanghai Disneyland from the three dimensions of tourists’ basic attributes, personality types, and interests, which can make the marketing of the scenic spot more targeted and increase the utilization rate of resources in the scenic spot. Yuan et al.^43^ modelled tourist behaviour characteristics and preferences based on various tourist data for Wudang Mountain scenic spots, explored developing local tourism and improved the tourist experience.

Based on previous research, analysing evacuation issues in different scenarios should be different, and in analysing tourist evacuation problems, priority should be given to the tourist experience. The effectiveness and willingness to cooperate in evacuation routes should be improved by utilizing tourists’ tourism preferences and route complexity. In addition, it is necessary to study the visualization system of evacuation routes to further improve the monitoring and management of tourist flow in scenic spots.

## The proposed evacuation route recommendation method

### The preference modelling of tourism and scenic spots based on a GNN

First, the tourist-scene interactive data are vectorized, and the embedded vector $e_v\in R^{dim} (e_s\in R^{dim} )$is used to describe the tourist *v* and scenic spot *s*, where *dim* represents the size of the embedded dimension.

1 $$\begin{aligned} E=[\underbrace{e_{v_1 },\cdots ,e_{v_N }}_{tourists\ embeddings},\underbrace{e_{s_1 },\cdots ,e_{s_M }}_{scenic\ spots\ embeddings} ] \end{aligned}$$

The main idea of the preference model of tourism and scenic spots based on GNN is to build a high-level interaction diagram based on the interaction relationship between tourists and scenic spots, encode the interaction information into the embedding vector, and then generate a high-level embedded communication layer, embedded aggregation layer and prediction layer to calculate the prediction score of tourists and scenic spots to be recommended. A 3-layer GNN model is shown in 1.

**Figure 1:**
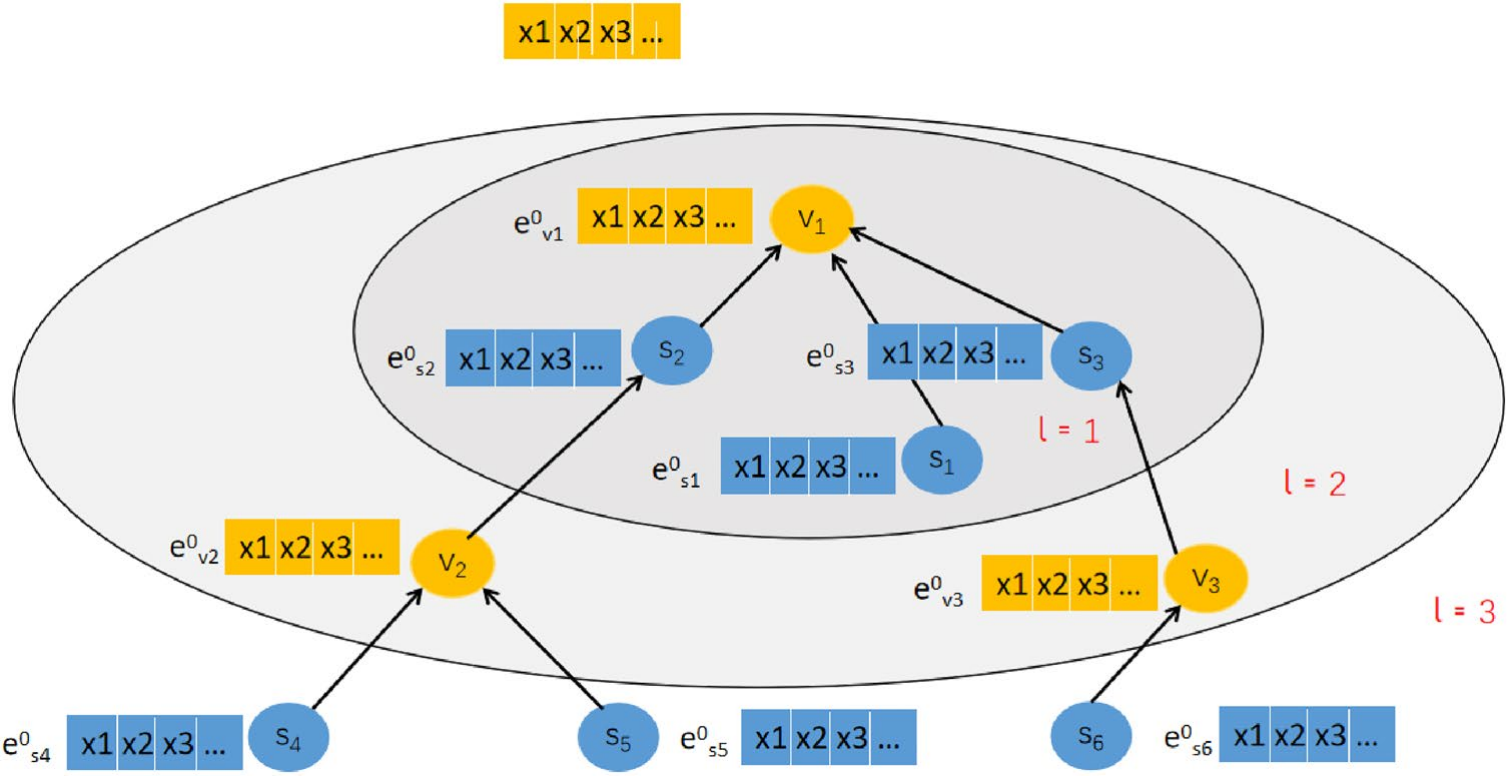
An illustration for high-order interactive information of the tourists and scenic spots based on GNN.

**Figure 2:**
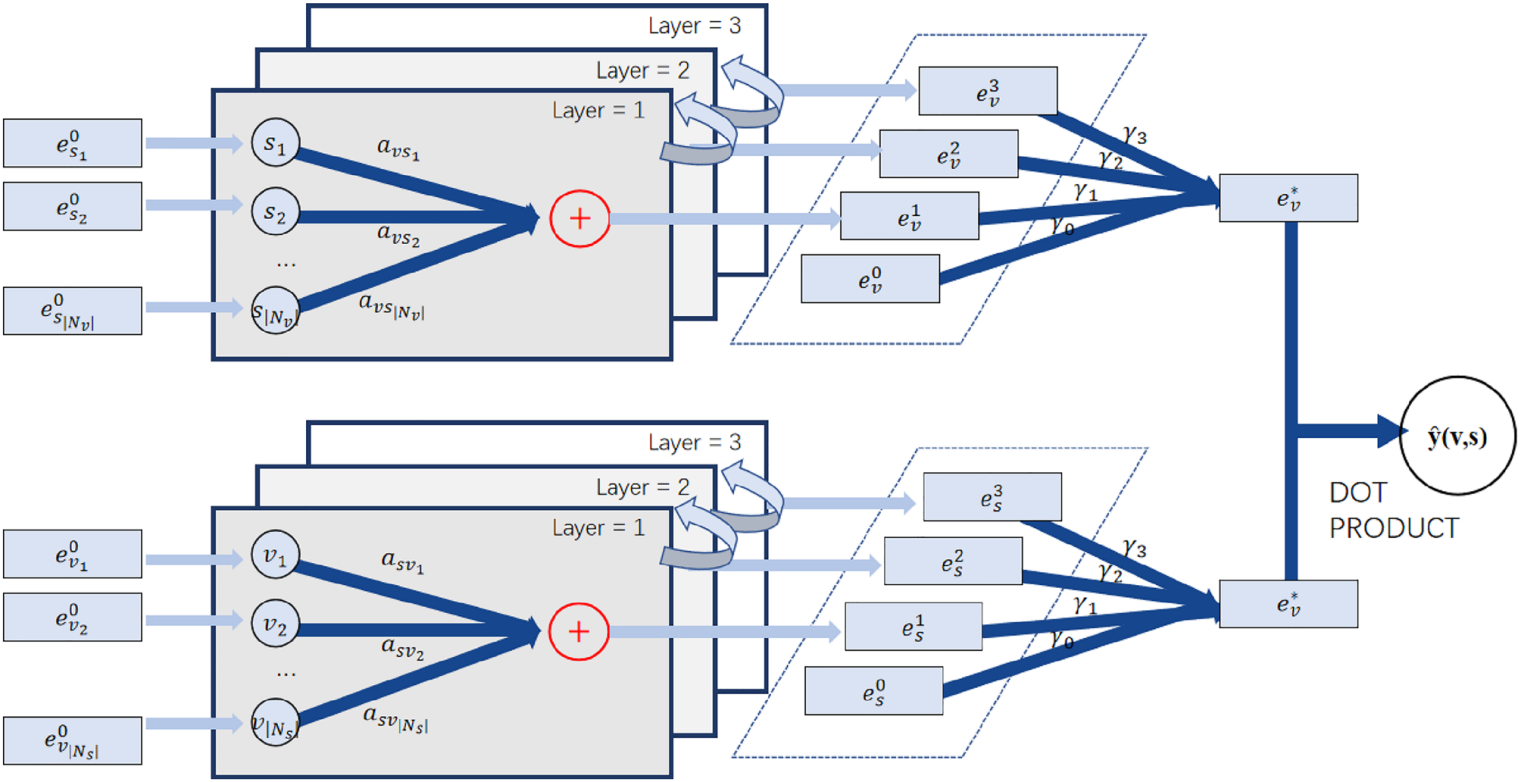
An illustration of GCN model.
  Then, the embedded information of each order is aggregated into a single vector through the aggregation function, which is defined as follows:
  5 $$\begin{aligned} e_{v}^{*}= & {} {\textstyle \sum _{l=0}^{L}} p_le_{v}^{(l)} \end{aligned}$$
  6 $$\begin{aligned} e_{s}^{*}= & {} {\textstyle \sum _{l=0}^{K}} p_le_{s}^{(l)} \end{aligned}$$
  where, $$p_k=\frac{1}{(L+1)} $$.
  Finally, the aggregate embedded representation of tourists *v* and scenic spots *s* are calculated by inner product to calculate the predicted score ${\hat{y}} (v,s)$ of tourists *v* on scenic spots *s*:
  7 $$\begin{aligned} {\hat{y}} (v,s)=e_{v}^{* \top }e_{s}^{*} \end{aligned}$$
  In order to optimize the GNN recommendation model, the Bayesian Personalized Ranking (BPR) loss function is selected. BPR calculates the overall loss of the model by assuming that tourists should have a higher predictive value for the scenic spots they have visited than for the scenic spots they have not visited.
  8 $$\begin{aligned} L_{BPR}=- {\textstyle \sum _{(v,i,j)\in O}}\ln {\sigma ({\hat{y}}_{(v,i)}-{\hat{y}}_{(v,j)} )} +\lambda \left\| \Theta \right\| ^2 \end{aligned}$$
  where $O=\left\{ (v,i,j)\mid (v,i)\in R^+,(v,j)\in R^- \right\}$ represents the training set, $R^+$ represents the scenic spots that tourists have been visited, $R^-$ is the scenic spots that tourists have not been visited through random negative sampling strategy; $\sigma$ is the sigmoid function; $\lambda$ is used to control *L*2 regularization intensity, $\Theta$ represents the parameters of the model as a whole, that is, the initial embedding vector. We use the mini-batch adam optimizer to optimize and update the model parameters.

1. High-level embedded communication layer
  As shown in Fig. 1, the scenic spots *s* related to tourist *v* are encoded as first-order embedding information of tourist *v* to enhance the embedding information of tourist *v*. The definition is as follows:
  2 $$\begin{aligned} e_{v}^{(l)} = {\textstyle \sum _{c\in N_v}^{}} \alpha _{vs}e_s \end{aligned}$$
  where $e_{v}^{(l)}$ is the first-order connectivity information of tourist v, $e_s$ represents the initial embedding of scenes; $\alpha _{vs}$ represents the attenuation coefficient of each propagation on the *edge*(*v*, *s*), gradually attenuating with the change in route length during the embedded propagation. In this paper, we adopt the same idea as neural graph collaborative filtering (GCN)^16^, assuming that $\alpha _{vs}=\frac{1}{(\sqrt{\left| N_v \right| } \sqrt{\left| N_s \right| } )}$,where $\left| N_v \right|$ and $\left| N_s \right|$ indicate the number of first-hop neighbours of tourist *v* and scene *s*, respectively.
  According to the transmission form of the first-order embedded information, *l*-order (higher-order) embedded information of tourist *v* is obtained as follows:
  3 $$\begin{aligned} e_{v}^{(l)}= {\textstyle \sum _{c\in N_v}} \frac{1}{(\sqrt{\left| N_v \right| } \sqrt{\left| N_s \right| } )}e_{s}^{(l-1)} \end{aligned}$$
  In the same way, the expression form of the *l*-order (higher-order) embedded information of scenic spots *s* as follows:
  4 $$\begin{aligned} e_{s}^{(l)}= {\textstyle \sum _{u\in N_s}} \frac{1}{(\sqrt{\left| N_s \right| } \sqrt{\left| N_v \right| } )}e_{v}^{(l-1)} \end{aligned}$$
2. Embedded aggregation layer
  As shown in Fig. 2, after the embedded propagation of layer *l*, the high-level information$\left\{ e_v^{(0)},\cdots , e_v^{(L)} \right\}$ of tourist *v* and $\left\{ e_s^{(0)},\cdots , e_s^{(l)} \right\}$ of scenic spots *s* are obtained.

### Preference prediction score based on tourism style similarity

Historically visited scenic spots not only represent tourist preferences but also show a similar tourism style characteristic. This paper uses the similarity calculation of tourism style to improve the prediction accuracy of tourist preference recommendations and thus increases the willingness of tourists to cooperate in evacuation management.

1. Definition of scenic spot style
  Assume that the style of the scenic spot is represented by the real value vector Ss from 0 to 1, where (his, mus, art, par, net, rel, mod) represent seven tourism styles of historical buildings, museums, art galleries, parks, natural landscapes, religious buildings, and modern landscapes, respectively. The high value proves that this style of the scenic spot is more obvious. The style of the scenic spot is expressed as follows:
  9 $$\begin{aligned} S_s=(his,mus,art,par,net,rel,mod) \end{aligned}$$
  Taking the “Pinghu Autumn Moon”, “Jindai Bridge” and “Zhejiang Provincial Museum” in the West Lake scenic spot as example, the scenic spot-style vectors are shown in Table 1.

**Table 1:** Examples of scenic spot style vectors.

scene	his	mus	art	par	net	rel	mod
Pinghu autumn moon	0.92	0.18	0.19	0.35	0.75	0.02	0.22
Jindai bridge	0.90	0.15	0.12	0.09	0.75	0.10	0.50
Zhejiang provincial museum	0.54	0.97	0.41	0.31	0.36	0.18	0.82
$\cdots$	$\cdots$	$\cdots$	$\cdots$	$\cdots$	$\cdots$	$\cdots$	$\cdots$
2. Definition of tourism style
  The tourism style $S_v$ is defined as the statistical result of the style of scenic spots visited in history, calculated as follows:
  10 $$\begin{aligned} S_v=\frac{ {\textstyle \sum _{p=1}^{P}}(his,mus,art,par,net,rel,mod)}{P} \end{aligned}$$
  where *P* is the number of scenic spots visited in history. Finally, the tourism style of each tourist can be calculated, as shown in Table 2.

**Table 2:** Examples of tourism style vectors.

tourists	his	mus	art	par	net	rel	mod
tourist1							
tourist2							
tourist3							
$$\cdots $$
3. Calculation method of tourist style similarity.
  Between scenic spot and tourism styles, and the similarity score $$Sim_{vts}(v,s) $$ is shown below: Based on the scenic spot-style vector and the tourism style vector, the cosine similarity is used to calculate the similarity.
  11 $$\begin{aligned} Sim_{vts}(v,s)=\frac{ {\textstyle \sum _{i=1}^{n}}(S_{v_i}\times S_{s_i}) }{\sqrt{ {\textstyle \sum _{i=1}^{n}(S_{v_i})^2} }\times \sqrt{ {\textstyle \sum _{i=1}^{n}}(S_{s_i})^2 } } \end{aligned}$$
  where, $$ S_{v_i}$$ represents the *i*th value in the tourism style vector and $$S_{s_i}$$ represents the *i*th value in the scenic spot style vector.
  Then, sum the predicted score ${\hat{y}}(v,s)$ obtained in “Preference prediction score based on tourism style similarity” section and the tourism style similarity score $Sim_{vts} (v,s)$ in this section by weighting them and obtain the improved scoring model as follows:
  12 $$\begin{aligned} F(v,s)=(1-k){\hat{y}}(v,s)+kSim_{vts}(v,s) \end{aligned}$$
  where *k* is the weight.

### Optimal tourist evacuation route design considering route complexity and real-time carrying capacity of scenic spots

1. Calculate evacuation route complexity
  In the above steps, we consider tourists’ tourism style to enhance their willingness to cooperate. In the following steps, we further improve evacuation efficiency and effectiveness. The evacuation route complexity is one of the important factors that affect evacuation efficiency. This paper measures evacuation route complexity by calculating the distance from the recommended scenic spots (evacuation destinations). The longer the distance is, the lower the evacuation efficiency. The route complexity calculation method from scenic spot (starting point $x_i$) to scenic spot (destination $y_j$) is as follows:
  13 $$\begin{aligned} Dis(x_i,y_i)=\frac{Dis(x_i,y_i)}{Dis_{max}} \end{aligned}$$
  where, $i=\left\{ 1,2,\cdots ,n \right\}$, $j=\left\{ 1,2,\cdots ,n\right\}$,n is the number of scenic spots, and $Dis_{max}$ is the longest distance among all the scenic spots.
2. Real-time carrying capacity calculation method
  To avoid new congestion at evacuation destinations, the real-time carrying capacity of the scenic spot is also an important factor to be considered. Assuming that the total capacity of the scenic spot is $a_i$ and the current number of people in the scenic spot is $r_{cur}$, the real-time carrying capacity $wr_i$ of the scenic spot is calculated as follows:
  14 $$\begin{aligned} wr_i=\frac{a_i-r_{cur}}{max(a_i-r_{cur})} \end{aligned}$$
  where $i=\left\{ 1,2,\cdots ,n \right\}$, *n* is the number of scenic spots, and $max(a_i-r_{cur})$ is the maximum real-time bearing capacity of all scenic spots. In summary, the tourist evacuation route recommendation method based on the graph neural network proposed in this paper consists of three parts: the scenic spot recommendation algorithm based on the graph neural network (Part A of “The proposed evacuation route recommendation method” section), tourist style similarity calculation (Part B) and calculating route complexity and real-time carrying capacity of scenic spots (Part C). The final evaluation score for tourist *v* and scenic spot *s* is calculated as follows:
  15 $$\begin{aligned} FS(v,s)=F(v,s)+PP*Dis(x_i,y_j)+PR*wr_i \end{aligned}$$
  where *PP* is the weight of route complexity and *PR* is the carrying capacity weight. In “Visualization analysis results” section, the parameter settings of *PP* and *PR* parameter settings will be visually analysed.

## Introduce the design method of the visualization system

**Figure 3:**
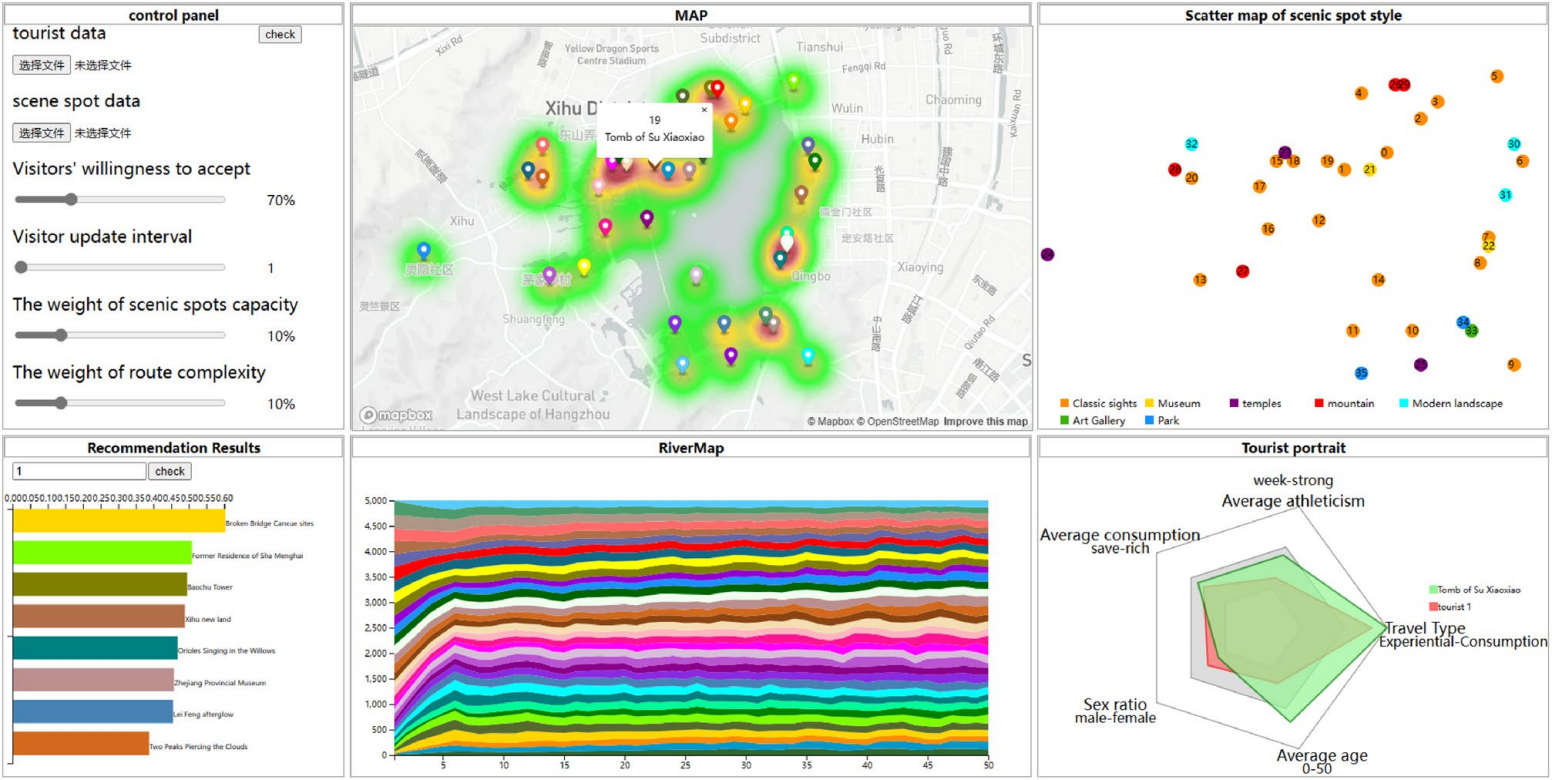
The design of the scenic spot visualization system.

In this paper, the visualization system design for scenic spot evacuation management is based on D3. JS: The functional visualization system design includes six parts: control panel design, map visual interface design, scenic spot-style clustering visual design, scenic spot recommendation result in visual design, scenic spot evacuation efficiency visual design, and tourist portrait visual design, shown in Fig. 3.

### Control panel

The data format includes .xlsx and .txt files, and the content of the data file is the historical interactive data of tourists and scenic spots. The control panel parameter design includes tourists’ willingness to cooperate. Adjustable parameters are set to 60%, 75%, 90%, and 100%; the tourist update interval is set to [1,10]; the weight of scenic spot capacity is set to [0,0.5]; and the weight of route complexity is set to [0,0.5].

### Cartographic visualization (MAP)

The map data are an interactive map generated by Map Box GL JS, which comes from a front-end JavaScript library. Considering the carrying capacity of each scenic spot, the location colours are light green, dark green, yellow, orange and red, based on the number of tourists from low to high. When the number of tourists reaches 600, the colour is light green. . After that, the colour depth increases by one level for each increase of 60.

### Scenic spots style visualization (scatter map)

Using “classic scenic spots”, “museums”, “temples”, “mountains”, “modern landscapes”, “art galleries”, and “parks” as evaluation indicators, we obtain the style score of each scenic area and then construct a scenic area style vector. Based on that, the K-means method (K = 7) is used to obtain clustering information. In the Fig. 3(3), different colours are used to show the clustering results of each scenic spot and show the correlation of different attractions. Each clustering scatter represents a scenic spot; when it is clicked, the scenic spot name, scenic style and tourist portrait can be displayed in Fig. 3(6).

### Recommendation result visualization

To visually display the recommendation results for different tourists (by entering the tourist’s ID), we analyse the impact of parameter settings on the recommendation results. First, the tourist and scenic spot data should be imported through the control panel. The ‘attraction capacity weights (SC)’ and ‘route complexity weights (RC)’ can be adjusted in the control panel. Then, according to the proposed method in “The proposed evacuation route recommendation method” section, the preference score of scenic spots for different tourists is calculated and sorted. Finally, ranking the attraction recommendation results is shown in Fig. 3(4), and the horizontal axis represents the recommended rating of each scenic spot.

### Visualization of evacuation efficiency in scenic spots (RiverMap)

To visualize the evacuation efficiency of scenic spots, we adjust the ‘population weight’ and ‘route weight’ in the control panel and display the changes in the number of people in each batch through river maps. To display the evacuation effect, we set 10 rounds of action (moving or staying) for each batch, and the horizontal coordinates of the river map represent the total number of evacuations (evacuation total = batch * rounds, where we set the number of rounds to 10). Different colours represent different scenic spots, and the width represents the number of people in the scenic spots after the corresponding evacuation batch.

### Visualization of tourist portrait

Based on historical travel behaviours, tourists’ portraits can be visualized and analysed. First, we collected the tourists’ data, including age, gender, consumption level, sporting ability, and historical visited scenic spots. For each scenic spot, the tourists’ characteristics data are calculated and shown in radar charts. For each tourist, his or her portrait can also be shown in radar charts. Figure 3(6) shows a tourist portrait with ID 1. The tourist portraits of different scenic spots can also be shown in Fig. 3(6) by clicking on different scenic spots in Fig. 3(3).

## Experimental results and visualization analysis

### Data source description and visualization tasks

This paper takes West Lake as an example to verify the effectiveness of the proposed method. The experimental data were provided by the West Lake administration, including the real-time number of tourists and the registration data of tourists when they enter the park, including basic tourist information, historical visit records and satisfaction evaluations. After data cleaning, a total of 5000 available data points were obtained. The above data were used to test the effectiveness of the tourist evacuation route recommendation method proposed in this paper and to perform the following visualization tasks.

1. Visualization analysis of real-time carrying capacity warning for the West Lake scenic area.
2. Visualization analysis of the style clustering results of West Lake scenic spots.
3. Recommendation result analysis of evacuation routes for tourists in the West Lake scenic area.
4. Visualization analysis of the evacuation efficiency of West Lake scenic spots (by adjusting the PP and PR parameters).
5. Visualization analysis of tourist portraits of West Lake scenic spots.

### Comparison experiments

To verify the effectiveness of the proposed PER-GCN in “The proposed evacuation route recommendation method” section, the PER-GCN algorithm is compared with the GCN (original algorithm) and collaborative filtering algorithm based on users (uCF) (classical algorithm) for comparison experiments. The experiments are run 30 times under an NVIDIA GeForce GTX 1060 5 GB environment. All algorithms are cross validated on the West Lake dataset with ten iterations, and the mean and variance are taken as the final experimental results of each algorithm with the precision rate and recall rate as the evaluation indices. The statistical results are shown in Table 3.

**Table 3:** Experimental results comparison.

Evaluation indicators	Precision			Recall		
Comparison algorithm	uCF	GCN	**PER-GCN**	uCF	GCN	**PER-GCN**
Average	59.935	65.579	**71.673**	48.671	50.42	**58.47**
Variance	0.48447	0.12638	0.15966	0.14613	0.14387	0.14264

Significant values are in bold.

To further test the significance of the comparison results, we used the pairwise (one-sided) Wilcoxon’s signed-rank test with a significance level $\alpha$ = 0.05 to compare the PER-GCN with uCF and GCN. The test results are shown in Table 4.

**Table 4:** Wilcoxon’s signed-rank test was performed with a significance level of $\alpha$ = 0.05.

*p* value	PER-GCN versus uCF	PER-GCN versus uCF
Precision	3.0142e−11	3.0104e−11
Recall	3.0066e−11	2.9953e−11

The results in Tables 3 and 4 show that the PER-GCN is significantly better than the GCN and uCF. The reasons are analysed as follows.

1. Although uCF is a classical method, it also has an obvious sparsity problem. With data scale expansion, the proportion of data rated by users in the overall database decreases, the sparsity of the user-rating matrix increases and becomes more serious, leading to significant decreases in accuracy when calculating the nearest neighbours of users or items, thus making the recommendation quality of the recommendation system drop sharply and the accuracy of mining information insufficient.
2. GCN uses the matrix decomposition technique, which is one of the solutions to the sparsity problem. The method expresses the relationship between nodes with the adjacency matrix and then decomposes the matrix to obtain the required embedding vector. Graph embedding represents the nodes in the graph as low-dimensional, real-valued, dense vector forms so that the obtained vector forms can represent and reason in the vector space; such vectors can be used in specific downstream tasks. Representation of the entire graph as a low-dimensional, real-valued, dense vector form is used to classify the entire graph structure. This algorithm reasonably increases the effectiveness of the algorithm. The recommendation algorithm based on GCN performs a secondary fusion operation on the existing results of GCN again. A simple dimensionality reduction method is adopted to set conditions to remove some users who have not participated in the rating or have rated very few times to reduce the dimensionality of the user-rating matrix and obtain a more accurate user-rating matrix. Then, the user-rating matrix was added to the model for fusion for a second time to further improve result accuracy.
3. Based on the advantages of the GCN, our method further considers the similarity of tourism styles. It uses the GCN to calculate tourists’ preferred scenic spots based on their historical travel routes, and then mining a list of scenic spots with similar styles through tourism style similarity calculation will further obtain tourists’ preference information. The GCN-PER algorithm will help alleviate cold start and sparsity issues, thus improving the recall and accuracy compared to the GCN algorithm.

### Visualization analysis results

#### The real-time bearing capacity warning of the West Lake scenic spot

**Figure 4:**
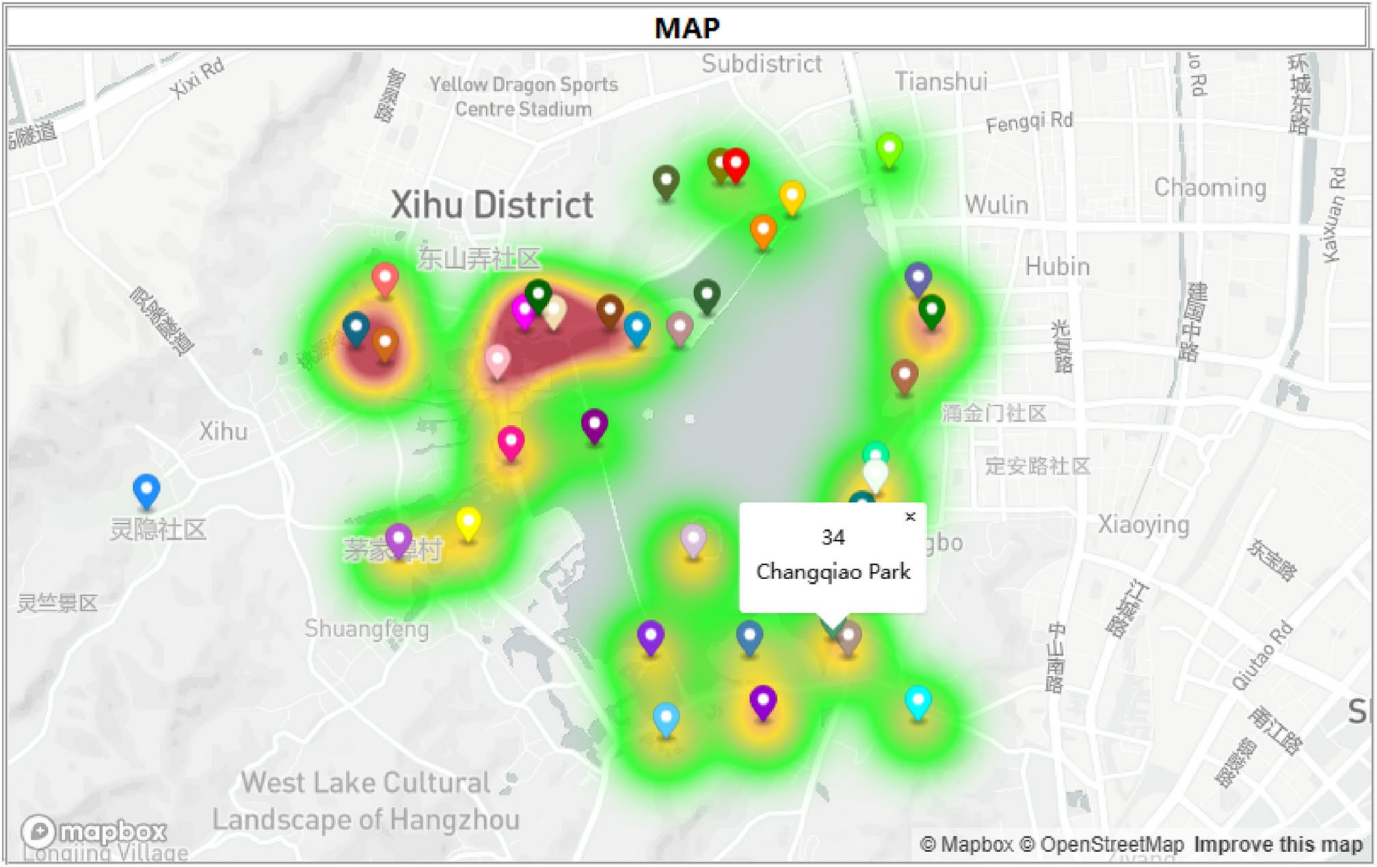
The visualization result for West Lake’s real-time carrying capacity.

In the visualization system, different colours are used to show the tourist flow and travel style of the scenic spots. For tourist flow, light green, dark green, yellow, orange and red were used to show the real-time tourist density. The colour from light green to red with tourist density increased, as shown in Fig. 4. For example, real-time carrying capacity warning shows that the tourist flow of Zhejiang Art Museum, Qu Yuanfeng, and Yuewang Temple scenic spots exceeds the carrying capacity. The real-time carrying capacity warning is displayed in red, while there are fewer tourists from Wansong Academy and Jingci Temple with similar travel styles. In our proposed method, tourists to the Zhejiang Art Museum, Qu Yuanfeng, and Yuewang Temple scenic spots are recommended to go to Wansong Academy and Jingci Temple. For each scenic spot in Fig. 4, clicking on a certain scenic spot will display its ID and name.

#### Visualization analysis of the clustering results of scenic spot styles

**Figure 5:**
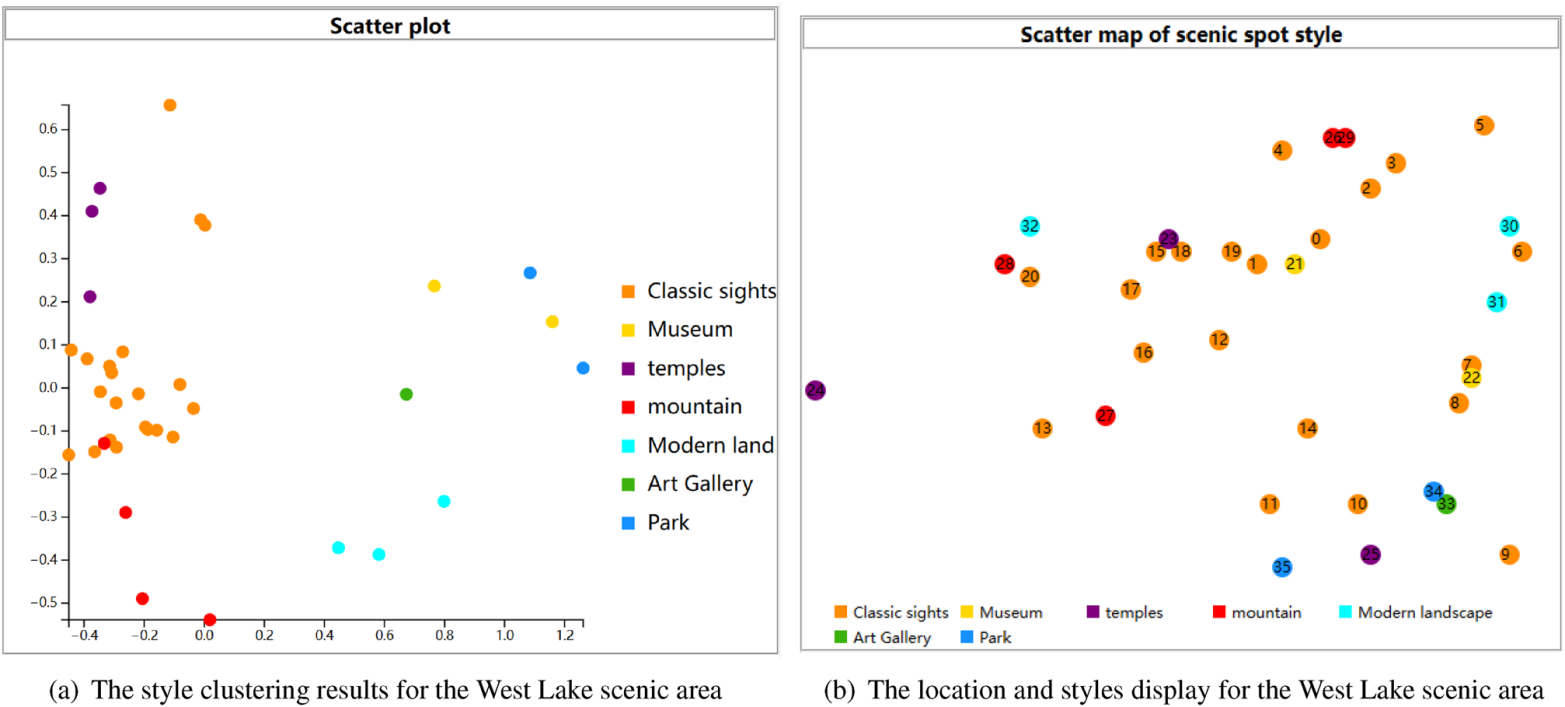
The visualization results of tourism style in the West Lake scenic area.

According to the method introduced in “Experimental results and visualization analysis” section, K-means was used to obtain clustering results for 36 scenic spots in West Lake, as shown in Fig. 5a. Based on the style scores of historical buildings, museums, art galleries, parks, natural landscapes, religious buildings, and modern landscapes, the location of 36 scenic spots and their style are displayed in the scatter plot shown in Fig. 5b. The seven types of scenic spots (classic sights museums temples mountains modern land art gallery park) are represented in orange, yellow, purple, red, cyan, green, and blue, respectively. The colours are clear between each category, and the difference is obvious so that tourists can easily observe the distribution of similar scenic spots. It is also allowed to select any style of attraction on the map to view tourist portraits.

The information extracted from the clustering results of the tourism style in the West Lake scenic area is used to obtain the style feature information and make labels for the scenic spots; then, using the scenic spot labels to mine the traveller’s preferred travel style, it can be found that Qu Yuan Feng He and Wan Song Shu Yuan, Yue Wang Temple and Jing Ci Temple have similar styles. Therefore, when the number of tourists in popular scenic spots exceeds the carrying capacity, the other scenic spots with similar styles will be recommended. Compared to the limited access and traditional evacuation methods, the proposed personalized recommendation method based on tourist preference can bring a better travel experience.

#### Visualization analysis of recommendation results based on different parameter settings

By adjusting the control parameters PR and PP (PR is the weight of route complexity, PP is the weight of scenic spot carrying capacity) in Equation (15), the recommended results of 36 scenic spots in West Lake are analysed.

**Figure 6:**
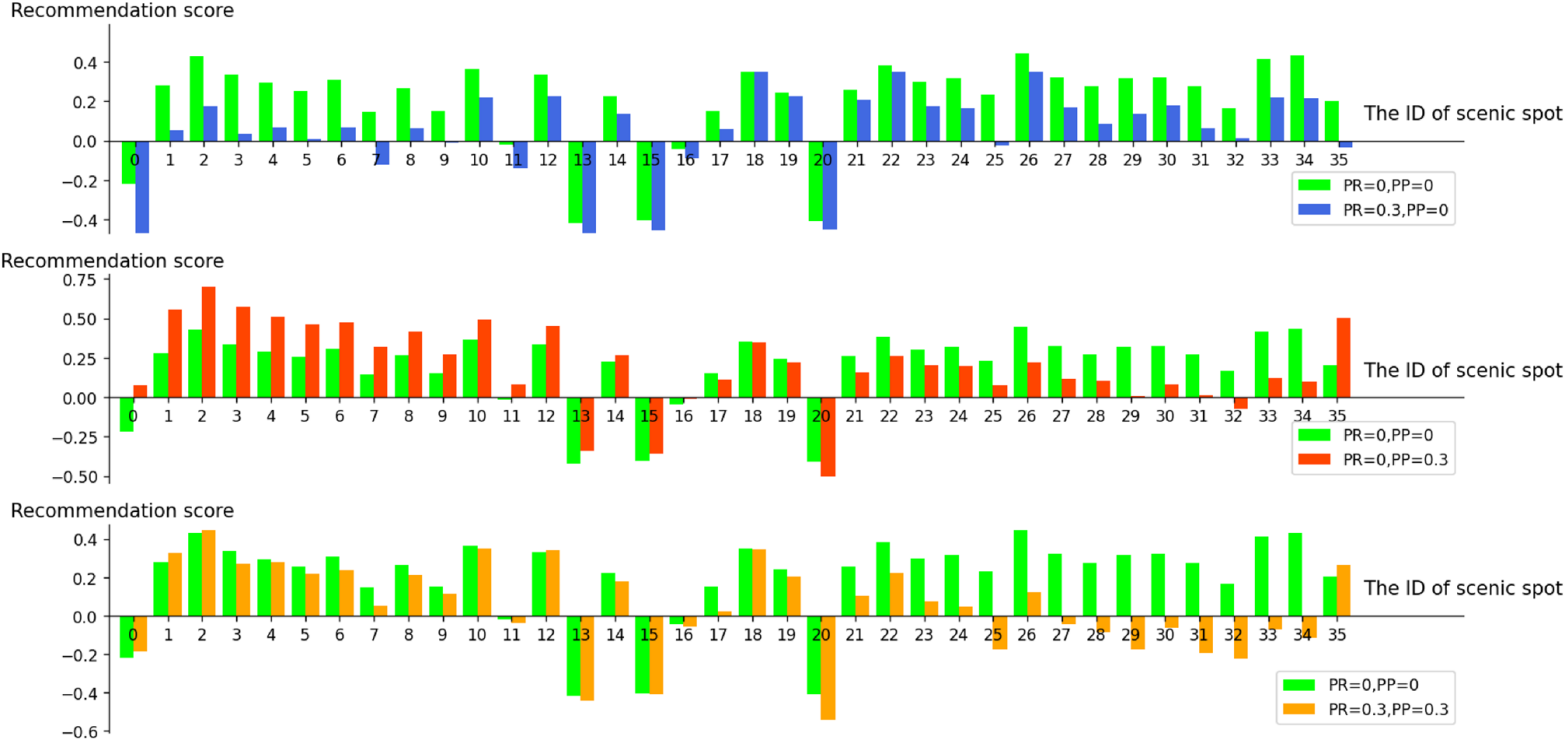
The recommendation results based on different PP and PR settings.

Figure 6 shows three bar charts displaying the recommendation scores for each scenic spot by adjusting PR and PP and comparing them with PR = PP = 0. When PR and PP are set to 0, only tourist preferences are considered to calculate the recommendation score. When PR is increased, the recommended score further considers the route complexity, and when PP is increased, the recommended score further considers the carrying capacity. Figure 6 shows the recommendation score for a tourist located in a scenic spot with ID 18 (Leifeng Xizhao). When PR = PP = 0, the scenic spot with ID 35 (Lingyin Temple) has a high recommended score (see green bar), but when PR is set to 0.3, the recommended score is significantly decreased (see blue bar). The reason is that Lingyin Temple is far from Leifeng Xizhao, which can be found in Fig. 5b. When PP = 0 and PR = 0.3, the scenic spot with ID 35 (Lingyin Temple) has a higher recommended score (see red bar). The reason is that Lingyin Temple has a greater carrying capacity, which can be found in Fig. 4. Therefore, when PP and PR are set to 0.3, the recommendation results account for both route complexity and carrying capacity. Therefore, the height of the orange bar is between the heights of the blue and red bars; that is, the recommendation scores are in the middle of the two.

#### Visualization analysis of evacuation efficiency based on cooperation degrees and evacuation batches

**Figure 7:**
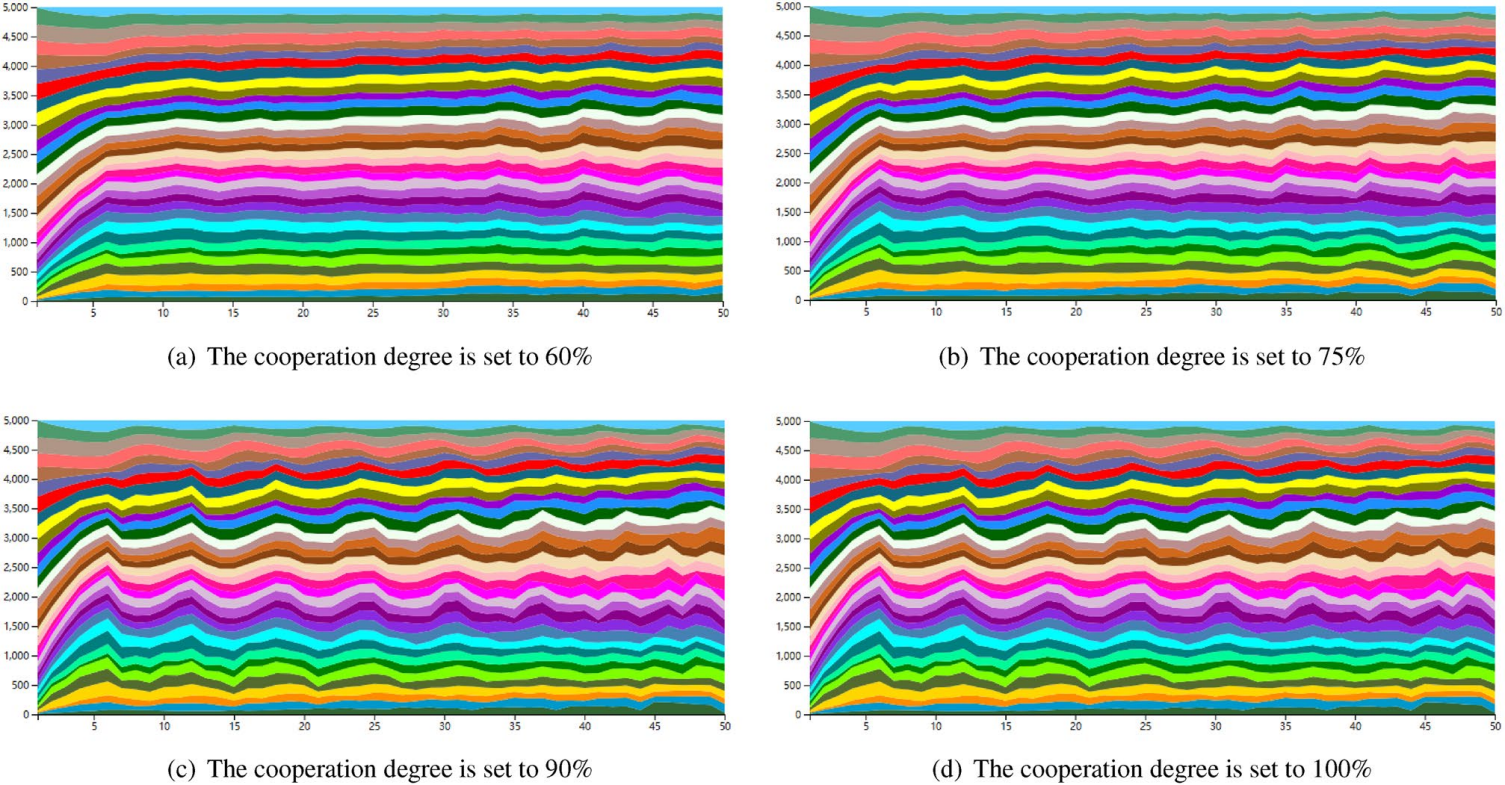
Changes in the river map with different settings of tourist willingness to cooperate.

**Figure 8:**
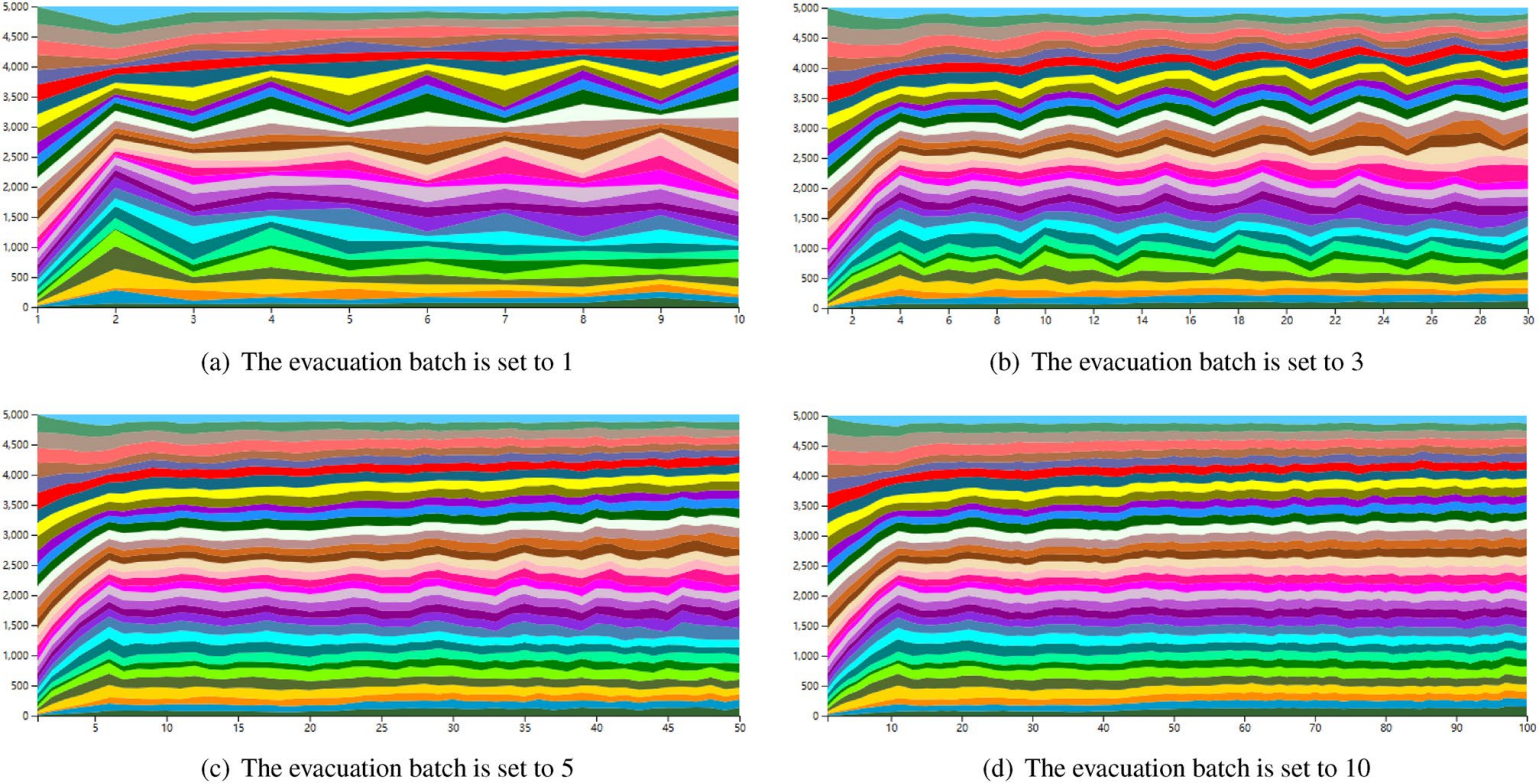
Changes in the river map with different evacuation batch settings.

When both PP and PR values are set to 0.1, the evacuation efficiency is analysed based on the willingness of tourists to cooperate and the evacuation batch, and a river map is drawn accordingly, as shown in Figs. 7 and 8.

As shown in Figs. 7 and 8 the higher the number of cooperating tourists (willingness to cooperate), the more unstable the river map; the fewer evacuation batches, the more unstable the river map. The reasoning is as follows:

1. The willingness to cooperate reflects, to a certain extent, the current flow of people during evacuation activities (willingness to cooperate * number of batches = number of people moving). However, when the willingness to cooperate is high, the flow of people fluctuates greatly, making the river map more unstable. Therefore, in our method, a certain number of tourists unwilling to cooperate can be tolerated, which is more in line with the actual situation and helps to improve evacuation efficiency.
2. For evacuation batches, it is equivalent to the update interval in the model. When the number of people is fixed, more batches indicate fewer people in a single evacuation activity, which can make the river map smoother and have fewer fluctuations.

Based on the above analysis, it can be concluded that the optimal parameters in the West Lake tourist evacuation recommendation system should be set as follows: when the willingness of tourists to cooperate is within the range of 60% -75%, and the batch setting is 10 or more times, the evacuation efficiency is higher.

#### Visualization analysis of tourist portraits in the West Lake scenic area

**Figure 9:**
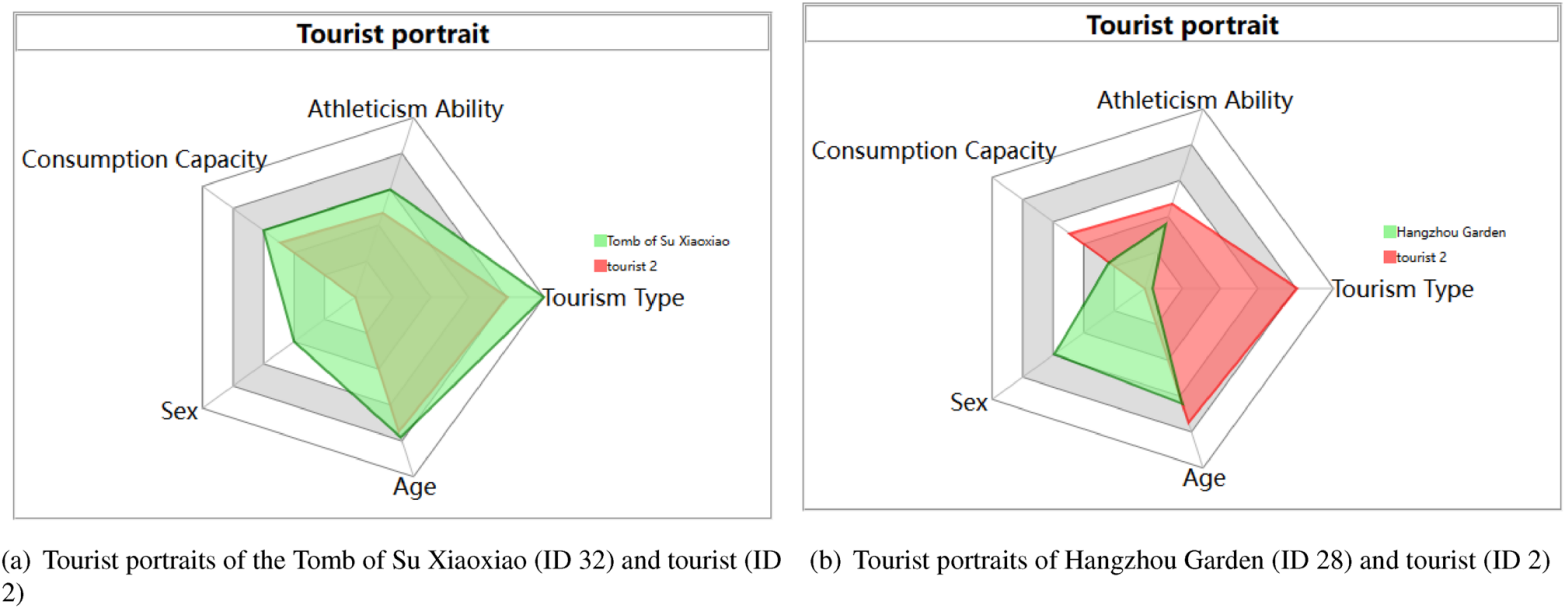
The tourist portraits of different scenic spots (taking the Tomb of Su Xiaoxiao and Hangzhou Garden as examples).

In this visualization system, radar chart is used to display the tourist portraits of each scenic spot and each tourist, as shown in Fig. 9. The tourist portraits can be switched to view all scenic spots and tourists by clicking the location (or ID) of the scenic spot in Fig. 3(2) or (3) and choosing a tourist ID number in Fig. 3(4).

In Fig. 9, there are five indicators to describe the tourist portrait, including sex, age, tourism type, athletic ability and consumption ability. The data come from tourists’ registration information and their historically visited scenic spots. In Fig. 9, the red area shows the tourist portrait of tourists, and the green area shows that of scenic spots, where the five scenic spot indicators are obtained by calculating the average value of tourist information that has visited the scenic spot. The range of sex is [0,1], where 0 represents male and 1 represents female. The age range is [15,65], and each grid represents a 10-year increase. The range of consumption capacity is [1000,6000], and each grid represents an RMB1000 increase. The consumption capacity refers to the acceptable travel expenses for tourists in West Lake. The range of athletic ability is [5000, 15,000], referring to the daily steps recorded in their mobiles. The scope of tourism type ranges from knowledge type to experience type, obtained from the style vectors of scenic spots and tourists in Table 1 and 2. Generally, the styles of the historical buildings, museums, art galleries, religious buildings, parks, natural landscapes, and modern landscapes are developed from knowledge type to experience type, and the value is from 1 to 0. Therefore, the highest score in each row of Tables 1 and 2 represents the tourism style of each scenic spot and tourist. Therefore, the tourist portrait of tourist (ID 2) is male, 53 years old, acceptable travel expenses are 3500, and the average daily steps are 9773. The tourism type is knowledge oriented. Based on that, the Tomb of Su Xiaoxiao and Hangzhou Garden tourist portraits can be analysed as follows:

1. The tourist portrait of “Su Xiaoxiao Tomb” has the following characteristics: the tourism type tends to be knowledge style, the tourists are relatively older in age, the majority are male, the consumption ability belongs to the luxury type, and athletic ability is moderate.
2. The tourist portrait of “Hangzhou Garden” has the following characteristics: the tourism type tends to be experience style, the age and gender are relatively average, the consumption ability belongs to the economic type, and athletic ability is relatively weak.

The visualization results of tourist portraits are beneficial for the West Lake administration to analyse the characteristics of scenic spots and tourists and provide specific customer services for different scenic spots.

## Summary and future work

This paper proposed an advanced recommendation algorithm based on GCN for tourist evaluation route planning (GCN-PER) and designed a visualization system for scenic spot administration. In tourist evacuation route planning, a graph neural network recommendation algorithm combining tourism style similarity calculation was proposed, improving recommendation accuracy and enhancing tourists’ willingness to cooperate. Based on that, the distance of recommended scenic spots and the real-time carrying capacity of each scenic spot were considered to optimize evacuation routes. Based on that, this paper took the West Lake scenic area as an example to visually analyse the characteristics of 5000 tourists and 36 scenic spots. This visualization system can assist the West Lake Management Administration in the real-time monitoring of scenic spots and use the GCN-PER algorithm for evacuation planning of crowded scenic spots, which is conducive to balancing the spatial and temporal distribution of tourists in various scenic spots. In addition, the visualization system designed in this paper can demonstrate the evacuation efficiency of scenic spots by different parameter settings.

There are also some limitations in this paper: (1) For tourists’ willingness to cooperate, although we conducted experiments on the impact of different willingness to cooperate on evacuation effectiveness, we only analysed the superiority of our method in theory (only by meeting tourists’ travel preferences can we attract more tourists to collaborate). In the future, we will further calculate the willingness of tourists to cooperate in practical applications and further optimize our evacuation model. (2) Based on the experimental results, we find that “the degree of willingness to cooperate” and “batch” have a great impact on the evacuation effect, but these parameters are only discussed by the visualization system. In the future, we will introduce parameter optimization models into this method to adaptively adjust the parameters.

## Data Availability

The datasets used and analysed during the current study available from the corresponding author on reasonable request.
